# Comparative Analysis of Low-Cost Portable Spectrophotometers for Colorimetric Accuracy on the RAL Design System Plus Color Calibration Target

**DOI:** 10.3390/s24248208

**Published:** 2024-12-23

**Authors:** Jaša Samec, Eva Štruc, Inese Berzina, Peter Naglič, Blaž Cugmas

**Affiliations:** 1 Vets4science d.o.o., 2 Kukovčeva Str., SI-3000 Celje, Slovenia; 2 Vetamplify SIA, 57/59—32 Krišjāņa Valdemāra Str., LV-1010 Riga, Latvia; eva@vetamplify.com; 3 VetCyto SIA, 13 Ozolu Str., LV-2008 Jurmala, Latvia; info@vetcyto.com; 4 Faculty of Electrical Engineering, University of Ljubljana, 25 Tržaška Str., SI-1000 Ljubljana, Slovenia; peter.naglic@fe.uni-lj.si; 5 Institute of Atomic Physics and Spectroscopy, University of Latvia, 3 Jelgavas Str., LV-1004 Riga, Latvia

**Keywords:** colorimetric accuracy, portable spectrophotometers, color calibration target, RAL Design System Plus, CIEDE2000 color difference, low-cost colorimetry, color constancy

## Abstract

Novel low-cost portable spectrophotometers could be an alternative to traditional spectrophotometers and calibrated RGB cameras by offering lower prices and convenient measurements but retaining high colorimetric accuracy. This study evaluated the colorimetric accuracy of low-cost, portable spectrophotometers on the established color calibration target—RAL Design System Plus (RAL+). Four spectrophotometers with a listed price between USD 100–1200 (Nix Spectro 2, Spectro 1 Pro, ColorReader, and Pico) and a smartphone RGB camera were tested on a representative subset of 183 RAL+ colors. Key performance metrics included the devices’ ability to match and measure RAL+ colors in the CIELAB color space using the color difference CIEDE2000 *ΔE*. The results showed that Nix Spectro 2 had the best performance, matching 99% of RAL+ colors with an estimated *ΔE* of 0.5–1.05. Spectro 1 Pro and ColorReader matched approximately 85% of colors with *ΔE* values between 1.07 and 1.39, while Pico and the Asus 8 smartphone matched 54–77% of colors, with *ΔE* of around 1.85. Our findings showed that low-cost, portable spectrophotometers offered excellent colorimetric measurements. They mostly outperformed existing RGB camera-based colorimetric systems, making them valuable tools in science and industry.

## 1. Introduction

Colorimetry quantifies and studies human perception of colors. If two objects are perceived as having the same color, they shall have the same colorimetric value [1]. Conversely, measured color differences should correlate with the visual color difference. Colorimetry is, thus, crucial in industrial quality control, where multiple batches of the same product are manufactured, for example, in textiles, paints, printing, and cosmetics [1].

Color perception depends on (1) a light source, (2) object properties affecting light reflectance or transmittance, and (3) an observer [2,3,4]. Predefined light sources, called standard illuminants, exhibit specified spectral power distribution. Some of the most popular standard illuminants [5] are the International Commission on Illumination’s (CIE) illuminants: A (related to a tungsten filament lamp), D50 (horizon light), and D65 (daylight). The CIE-defined observer (with a 2° or 10° visual field) is based on light-mixing and color-matching experiments [1]. Mathematically, perceived color is a sum of three tristimulus values: *X*, *Y*, and *Z*. Each tristimulus value is a product between a spectral color-matching function of the chosen standard observer ($\bar{x}(\lambda )$, $\bar{y}(\lambda )$, or $\bar{z}(\lambda )$), power distribution of the illuminant *S*(λ), and the object’s reflectance or transmittance spectrum *R*(λ).

The popular CIELAB color space transforms tristimulus values into coordinates L* (lightness), a* (opponent colors: red-green), and b* (yellow-blue), providing a more visually uniform color space [1]. L* varies between 100 (perfect white) and 0 (perfect black). The value of a* is positive for red and negative for green shades. Similarly, b* is positive for yellow and negative for blue colors. The first attempts to quantify the difference between two colors (Δ*E*) in the CIELAB color space were based on a simple Euclidian distance: ${\Delta E}_{CIE76}=\sqrt{\Delta L^{*2}+\Delta a^{*2}+\Delta b^{*2}}$. However, the CIELAB color space was less perceptually uniform than intended, particularly close to saturated values. Thus, the newest color differences formula of CIEDE2000 (*ΔE_00_*) addressed perceptual non-uniformities by replacing the a* and b* coordinates with chroma (C*) and hue (h*), and adding several corrections to better quantify human eye perception [6]. The human eye can spot color differences of 2.3–5.0, called the just-noticeable difference (JND) [7].

To determine a specific color, spectrophotometers acquire missing information, i.e., light reflectance or the transmittance spectrum. The spectrophotometer’s applicability, functionality, and portability are defined by its geometry, specified by the light source and observer’s angles. Most commonly, 45°/0° or 0°/45° geometry is used for matt surfaces due to excluded gloss [8]. An alternative for shiny surfaces is an integrating sphere spectrometer, which provides diffuse illumination and collects the light at an 8° angle (i.e., diffuse/8° or d/8° in short). Finally, multi-angle spectrophotometers are used for more complex surfaces or industrial requirements.

There are several portable spectrophotometers on the market. The scientific community often considers CM-700d (Konica Minolta KK, Tokyo, Japan) a reference when calibrating colorimetric systems or performing colorimetric measurements [9,10]. CM-700d includes the d/8° geometry, pulsed xenon lamp, and 31 light-collecting channels (10 nm steps between 400–700 nm). X-rite (Grand Rapids, MI, USA) is another popular manufacturer of portable spectrophotometers such as Ci64 (d/8°) or eXact™ (45°/0°). The listed inter-instrument agreement of these high-end state-of-the-art spectrophotometers is around 0.2 *ΔE_00_*. However, standard spectrophotometers are relatively expensive, with listed prices between USD 5000 and 10,000. Furthermore, they provide an average measurement at a single point, limiting their feasibility in the colorimetric study of an area.

**Figure 1:**
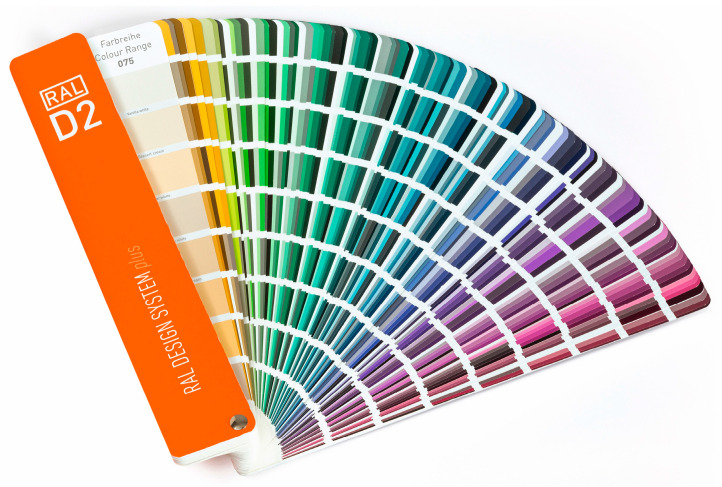
Color calibration target (CCT) RAL Design System Plus (©RAL gGmbH, Bonn, Germany, reproduced with permission from RAL gGmbH).

We can also colorimetrically calibrate commercial RGB cameras to achieve lower costs and spatial measurements. Color calibration relies on a model that maps measured (RGB) colors with the standard color space, such as CIELAB. The model is trained on color calibration targets (CCTs) or color reference charts, which can be in a single-page or fan format. The most popular CCTs are ColorChecker Classic (X-rite, Grand Rapids, MI, USA) [11,12], IT8 Targets (LaserSoft Imaging AG, Kiel, Germany) [13], RAL Design System Plus (RAL+, RAL gGmbH, Bonn, Germany, Figure 1) and Pantone (Pantone LLC, Carlstadt, NJ, USA). In biomedicine, various colorimetric systems with calibrated (smartphone) RGB cameras achieved colorimetric accuracy (*ΔE*) between 2.2 and 8.4 [11,12,14,15,16,17]. The color constancy of the existing systems is not optimal because most of these color differences could be spotted by the human eye [7].

The recently available low-cost (USD < 1200) and portable spectrophotometers could fill the gap between standard spectrophotometers (=high colorimetric accuracy) and RGB camera-based colorimetric systems (=affordability). Popular low-cost spectrophotometers, such as Color Muse (Variable Inc., Chattanooga, TN, USA), Nix Mini 2 or Pro (Nix Sensor Ltd., Hamilton, ON, Canada), and Cube (Palette Pty Ltd., Melbourne, Victoria, Australia), have been applied to the monitoring of food quality, environment, and radiation [18,19,20,21]. Compared to the standard spectrophotometers on food and soil, their colorimetric accuracy (*ΔE_00_*) was, on average, between 4.3 and 11.4 [20,21]. The color differences decreased to 2.1–6 and ~1.0–1.7 * (* our estimates) when low-cost spectrophotometers were tested on custom-made and RAL+ CCTs, respectively [22,23].

Most existing studies tested the colorimetric accuracy of low-cost spectrophotometers on non-standardized objects (food, soil, custom-made CCT). Furthermore, new versions of affordable devices like Nix Spectro 2 (Nix Sensor Ltd., Hamilton, ON, Canada) and Spectro 1 Pro (Variable Inc., Chattanooga, TN, USA) feature resolution with 31 channels and low inter-instrument agreements (*ΔE_00_* < 0.35), comparable to the standard spectrophotometers. Therefore, in this study, we tested the colorimetric accuracy of four new low-cost, portable spectrometers in matching and evaluating RAL Design System Plus (RAL+) colors. The results provided important insights into the performance of low-cost, portable spectrophotometers, especially compared to the custom-made colorimetric systems based on the calibrated RGB cameras. This study helps professionals and researchers in fields such as biomedicine, food hygiene, and environmental science find accessible but accurate tools for colorimetry.

## 2. Materials and Methods

### 2.1. Spectrophotometers

**Figure 2:**
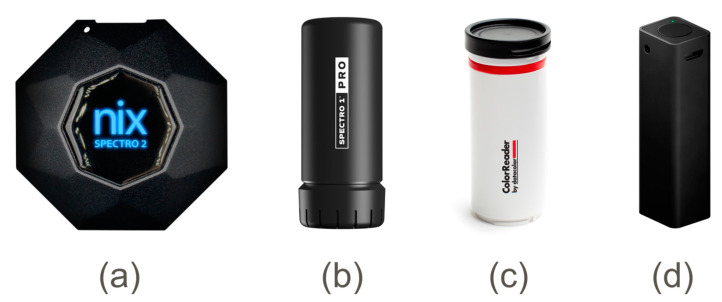
Spectrophotometers (**a**) Nix Spectro 2, (**b**) Spectro 1 Pro, (**c**) ColorReader, and (**d**) Pico ((**a**) ©Nix Sensor Ltd., Hamilton, ON, Canada; (**b**) ©Variable Inc., Chattanooga, TN, USA; (**c**) ©Datacolor GmbH, Marl, Germany; (**d**) ©Palette Pty Ltd., Melbourne, Victoria, Australia; images are reproduced with permissions from Nix Sensor Ltd., Variable Inc., Datacolor GmbH, and Palette Pty Ltd.).

This study enrolled four spectrophotometers (Table 1, Figure 2), covering a price range between USD 100 and 1200. The devices typically require Bluetooth connection with smartphones to display data and alter spectrophotometer settings. Smartphone applications provide details on colorimetric measurements like color values (e.g., CIELAB, sRGB, etc.) and the closest matches with the corresponding *ΔE* to standard CCT colors (e.g., RAL, Pantone). This study also included a smartphone Asus Zenfone 8 (ASUSTeK Computer Inc., Taipei, Taiwan) combined with a DermLite DL1 dermatoscope (DermLite LLC, Aliso Viejo, CA, USA) to guarantee stable illumination conditions [11].

### 2.2. Measurements

Colorimetrical accuracy was tested on RAL+ colors, which are labeled with seven-digit codes (HHH LL CC) representing hue (H), lightness (L), and chroma (C). RAL+ includes 1825 colors, covering most of the CIELAB color space (i.e., H: 0–360°, L: ~20–90, C: ~0–70). However, the RAL+ manufacturer provides only color light reflectance values(LRV) [24], indicating that the “real” RAL+ colors differ from their labeled CIELAB values. Therefore, some portable spectrophotometer manufacturers evaluated actual RAL+ color values independently, and their colorimetric assessments are sometimes directly available in smartphone applications that control the portable spectrometers.

For the sake of clarity, we employ the following terminology for the CIELAB values (L*, a*, b*) of RAL+ colors:

- *Labeled* values were calculated from the RAL+ color label (e.g., 010 60 15 translates to 60, 14.8, 2.6),
- *Assessed* values are the actual RAL+ color values, as evaluated by the portable spectrophotometer manufacturers (e.g., Spectro 1 Pro manufacturer foresaw 62.1, 14.3, 2.0 for the RAL+ color 010 60 15),
- *Measured* values were estimated in this study using portable spectrophotometers or a smartphone (e.g., 59.4 15.2 2.4 for the RAL+ color 010 60 15 by Nix Spectro 2 spectrophotometer),
- *Normalized* are measured values, normalized to *labeled* values (as defined in Section 2.4).

To rationalize measurements, we downsampled the number of enrolled colors to 183. By using random permutation and a 10-fold decrease (*randperm* function, Matlab R2017b, MathWorks, Natick, MA, USA), we retained the original RAL+ color distribution. Downsampling kept the differences in the L*, a*, and b* value histograms (10-unit wide bins) between entire and downsampled RAL+ colors below 1.1%. On the other hand, we acquired all 1825 RAL+ colors by the smartphone to prevent model overfitting when transforming measured colors into labeled ones (Section 2.4). We also excluded 27 smartphone measurements due to image saturation.

First, we calibrated the spectrophotometers according to the manuals. The color measurements were conducted in the ascending order of H, L, and C values in one continuous session. For each RAL+ color, we recorded (1) the *measured* CIELAB value, (2) the matched RAL+ color, and (3) *ΔE_00_* to the *assessed* RAL+ color (the Pico spectrophotometer did not provide *ΔE*).

### 2.3. RAL+ Color-Matching Accuracy

The *strict* RAL+ color-matching accuracy was calculated as a ratio between correctly matched and all enrolled colors (*n* = 183). For ColorReader, we excluded two faulty measurements from further analysis (*n* = 181). The Pico spectrophotometer did not offer color matching; thus, we selected the color with a minimal *ΔE_00_* between the *measured* and *labeled* RAL+ color values. The same procedure was applied to the smartphone. Since the Spectro 1 Pro did not display information on 19 (*assessed*) RAL+ colors, we additionally evaluated color-matching accuracies excluding these missing colors. On the other hand, Nix Spectro 2 and ColorReader presumably included all colors.

*Loose* color-matching accuracy additionally accepted the adjacent RAL+ color as the correct one. We defined an adjacent color as gradually changing in only one coordinate, i.e., hue, lightness, or chroma (for example, 020 30 30 vs. 030 30 30). These looser metrics are practical because most spectrophotometers match RAL+ colors according to their *assessed* and not *labeled* values. According to the Spectro 1 Pro database, the median difference between *labeled* and *assessed* RAL+ values is larger (*ΔE_00_* = 2.6) than between adjacent RAL+ colors (*ΔE_00_* = 2.1).

### 2.4. Color Difference

Color differences (*ΔE_00_*) were calculated between the *measured* and (1) *labeled* or (2) *assessed* RAL+ color values. However, we should note that the *labeled* RAL+ CIELAB color values are based on a particular measurement geometry. Therefore, the color difference between the *measured* and *labeled* colors cannot be a simple measure of spectrophotometer performance but rather an indicator of similarities between the device and RAL+ measurement geometry. Thus, we additionally normalized the *measured* against the *labeled* values and then calculated *ΔE_00_* between them.

Normalization was based on a regression model [11] estimating the relationship between the *measured* (*C_m_*) and *labeled* RAL+ CIELAB color values (*C_RAL_*):

*C*_*RAL*_ = *f*(*C*_*m*_), *C*_*RAL*_ ∈ {*L*_RAL_*, *a*_RAL_*, *b*_RAL_*} (1)

which served to normalize *C_m_*:

*C*_*m_norm*_ = *f*(*C*_*m*_). (2)

The spectrophotometers measured color values (*C_m_*) in the L*, a*, and b* coordinates, while the smartphone retrieved the RGB values. Potential overfitting on only 183 samples was avoided by (1) training and testing the model with a leave-one-out-cross-validation (LOOCV) approach and (2) choosing a simple linear function for *f*:

*C*_*RAL*_ = *p*_*0*_ + p_1_ L_m_* + p_2_ a_m_* + p_3_ b_m_*, *C_RAL_* ∈ {L_RAL_*, a_RAL_*, b_RAL_*}. (3)

The LOOCV approach enabled the same 183 RAL+ colors to be used for the regression model’s training and the color difference calculation between the *normalized* and *labeled* values.

On the other hand, the smartphone normalization model was trained on 1798 RAL+ colors (27 saturated images were excluded) but tested only on 183 selected colors. Since overfitting was not likely, a more complex polynomial was selected for *f*:

(4) $$C_{RAL}=\sum_{k=0}^{3}p_{k}C^{k}+p_{11}RG+p_{12}RB+p_{13}GB,\;C\in \left\{R,\;G,\;B\right\}$$

## 3. Results and Discussion

### 3.1. Color-Matching Accuracy

First, we evaluated the accuracy of four spectrophotometers and a smartphone in matching 183 RAL+ colors (Table 2). The *strict* color-matching accuracies ranged from 47.5 to 98.4%. Nix Spectro 2 performed the best with only three mismatched RAL+ colors. However, *strict* color-matching criteria could be too rigorous because the median difference between two random adjacent RAL+ colors (i.e., gradually different in only one coordinate) is smaller than that between the *labeled* and *assessed* RAL+ colors (see Section 2.3). Therefore, *loose* color-matching accuracy (i.e., adjacent RAL+ colors are also a correct match) can be considered a more practical measure.

With the loose approach, the color-matching accuracies significantly improved, ranging between 72.1% and 99.5%. Again, Nix Spectro 2 performed the best with almost perfect execution (99.5%). The only mismatched RAL+ color was opulent green, mistaken for intense green, which gradually differs in two coordinates (i.e., 160 20 20 vs. 170 20 15). Since the color difference (*ΔE_00_* = 3.74) between both *labeled* colors falls in the JND range [7], the color shades would probably be distinct to the human eye. Extrapolating its accuracy to the whole RAL+, Nix Spectro 2 would mismatch around nine RAL+ colors (out of 1825).

On the other hand, Spectro 1 Pro and ColorReader correctly matched ~86% of RAL+ colors. The misidentifications accounted for 25 colors (presumably ~250 in the whole RAL+ range). For these 25 mismatched colors, the median difference between the *assessed* and *measured* RAL+ colors was 4.1 for Spectro 1 Pro. The calibrated smartphone camera and Pico mismatched ~25% and ~45% RAL+ colors, respectively. We assume that a matching accuracy of around 75% can also be expected from the other calibrated smartphone- or camera-based colorimetric systems [12,14,16] since all tested smartphone cameras from our previous studies performed similarly [11,25]. Furthermore, Pico does not provide *assessed* colors. Thus, the color-matching accuracy was calculated according to the *labeled* RAL+ colors, which can be a contributing factor to an inferior performance.

We shall also alert readers to the proper selection of spectrophotometer settings for color matching. Nix Spectro 2 and Spectro 1 Pro enable different illuminants and observers. For example, when a different illuminant (D65 vs. D50) was selected for Nix Spectro 2, its *loose* matching accuracy dropped to only 88.0% (161/183), which is significantly worse than with the correct settings (Table 2). Additionally, metamerism may hinder a high-performing spectrophotometer from accurately matching the RAL+ color that visually aligns best with the unknown painted object.

### 3.2. Accuracy in ΔE_00_

The most straightforward approach to colorimetrically validate spectrophotometers would be based on the standardized color difference (*ΔE_00_*) between the *measured* and actual RAL+ color values. However, the actual RAL+ color values in the CIELAB color space remain unknown. Although the spectrophotometer’s color-matching capability (Section 3.1, Table 2) can be considered more of a qualitative than quantitative measure, these can serve to estimate an approximate colorimetric accuracy. With the almost perfect color-matching accuracy of the Nix Spectro 2 spectrophotometer, its absolute colorimetric accuracy shall be below *ΔE_00_* = 1.05, that is, 50% of the typical color difference between two RAL+ adjacent colors. Similarly, the equivalent estimate for Spectro 1 Pro was *ΔE_00_* < 1.19, based on 86.3% color matches and 13.7% mismatches exhibiting *ΔE_00_* of 4.1.

In order to verify these estimates further, we calculated median color differences (*ΔE_00_*) between the *labeled*, *assessed*, *measured*, and *normalized* RAL+ CIELAB color values as measured by four spectrophotometers and a smartphone (Table 3). The differences between the *measured* and *labeled* ranged between 1.27 and 3.95. The lowest color difference (*ΔE_00_* = 1.27) was provided by the ColorReader spectrophotometer, whose measurements seem adjusted to the *labeled* RAL+ color values the most. This hypothesis was confirmed by the fact that its color difference increased to 1.39 when the *measured* and *assessed* colors were compared. Expectedly, the detected color differences for Nix Spectro 2 and Spectro 1 Pro improved significantly to 0.52 and 1.07, respectively.

Our results showed better performance for the newly tested Nix Spectro 2, while for the other devices, they are consistent with a previous study [23], which reported a colorimetric accuracy of ~1.0–1.7* (*our estimates from *ΔE_CMC_*) for low-cost spectrophotometers. When devices were tested on visually heterogeneous objects (soil or food), *ΔE_00_* increased to 4.3–11.4 [20,21]. However, there were significant colorimetric disagreements of up to 3.8 even between standard instruments. With deducted inter-instrument color differences, the true colorimetric accuracy was probably lower, assumingly around 0.5–7.6, which is in the range of our results (Table 3).

However, relying solely on the differences between *measured*, *labeled*, and *assessed* colors does not allow for a colorimetric comparison with calibrated RGB cameras. Therefore, we also compared the *normalized* and *labeled* RAL+ colors, resulting in a *ΔE_00_* slightly above 1.1 for Nix Spectro 2, Spectro 1 Pro, and ColorReader, and around 1.85 for Pico and smartphone (Table 3(c)). Against the *measured–labeled* comparison (Table 3(a)), the color accuracy improved for around ~0.2–0.8, which is in line with the study of Kirchner et al. [23], where the normalization model improved the spectrophotometer colorimetric accuracy by ~0.7.

We shall stress that the smartphone’s median colorimetric accuracy *ΔE_00_* of 1.84 is one of the best reported. The other studies, including commercial smartphones or cameras, achieved a colorimetric accuracy above 2.2 [12,14,16]. The improved performance probably resulted from a complex calibration model trained on almost 1800 colors. Despite being effective, this approach was time-consuming and burdensome.

### 3.3. Overall Performance

The devices enrolled in this study exhibited three distinct colorimetric performances (Table 4). Nix Spetro 2 was the best-performing spectrophotometer, with almost perfect color matching and high colorimetric accuracy in evaluating RAL+ colors (*ΔE_00_* of mostly <1). On the other hand, it comes with the highest price, more than four times that of other spectrophotometers. Moreover, color matching requires a subscription, further widening the device price gap.

Spectro 1 Pro and ColorReader correctly matched around 85% RAL+ colors. However, absolute colorimetric accuracy (*ΔE_00_*) is probably slightly above 1. ColorReader could seem the obvious choice between these two devices due to its lower price, but the spectrophotometer stopped working shortly after our study, raising concerns about its durability and quality. On the other hand, Spectro 1 Pro included small magnets near the sensor opening to secure the protective cap. Because the magnets were installed slightly above the casing level, environmental light could penetrate onto firm surfaces like RAL+ sheets.

Pico and a smartphone camera exhibited similar performances—their median colorimetric error was just below JND, and, thus, undetectable by the human eye. Although smartphones are ubiquitous, proper colorimetrical calibration is time-consuming and burdensome, and the process demands add-ons to ensure stable illumination (dermatoscopes) and reference colors (color calibration targets). However, the most significant advantage of commercial (smartphone) cameras is spatial colorimetric imaging, which can reveal colors in smaller areas than the size of a typical spectrophotometer measurement aperture (e.g., the minimal diameter of Nix Spectro 2 opening is 2 mm).

## 4. Conclusions

In conclusion, our study indicated that the colorimetric performance of low-cost, portable spectrophotometers correlated with their price. Furthermore, cheaper devices (<USD 300) also performed very well, beating the colorimetric accuracy of a calibrated RGB camera. Therefore, instead of relying on custom-made optical setups, future biomedical studies on color measurement shall turn to commercial, low-cost, portable spectrophotometers to achieve optimal color constancy. The notable exception is when spatial measurements are needed. In the latter, color-mapping models shall be performed on numerous CCT colors.

## Figures and Tables

**Table 1:** Low-cost portable spectrophotometers evaluated in this study.

Spectrophotometer	Nix Spectro 2 ^a^	Spectro 1 Pro ^b^	ColorReader ^c^	Pico ^d^
Price (USD)	~1200	~300	~130	~120
Resolution	31 channels (10 nm steps between 400–700 nm)	31 channels (10 nm steps between 400–700 nm)	3 (RGB)	3 (RGB)
Geometry	45° (ring)/0°	Diffuse/0°	~35° (ring)/0° ^e^	~35°/~35° ^e^
Illumination	8 CRI LEDs	Full-spectrum LEDs	6 CRI LEDs	3 (LED) floodlights
Illuminants	A, C, D50, D55, D65, D75, F2, F7, F11	A, F2, D50, D65	/	/
Observer	2°, 10°	2°, 10°	/	/
Calibration steps	1	3	1	1
Claimed average inter-instrument agreement	0.35 *ΔE_00_*	0.35 *ΔE_00_*	/ ^f^	/ ^f^
Libraries	Several (incl. RAL, Pantone, NCS)	Several (incl. RAL, Pantone, NCS)	Several (incl. RAL, NCS)	Several (incl. RAL, NCS)

^a^ Nix Sensor Ltd., Hamilton, ON, Canada, www.nixsensor.com/nix-spectro-2/, ^b^ Variable Inc., Chattanooga, TN, USA, www.variableinc.com/spectro-1-pro.html, ^c^ Datacolor GmbH, Marl, Germany, www.datacolor.com/colorreader/products/colorreader, ^d^ Palette Pty Ltd., Melbourne, Victoria, Australia, palette.com.au/pico, ^e^ our estimation according to the illuminator/sensor geometry, ^f^ not found.

**Table 2:** Accuracies (in %) of four spectrophotometers and a smartphone in matching 183 RAL Design System Plus (RAL+) colors when (a) only studied colors (“*Strict*”), or (b) also adjacent colors (i.e., being gradually different in only one coordinate, i.e., hue, lightness, or chroma) were considered true (“*Loose*”).

Color-Matching Accuracy (%)	Nix Spectro 2	Spectro 1 Pro	ColorReader ^a^	Pico ^b^	Smartphone ^b^
(a) Strict	98.4	76.0 (86.3 ^c^)	78.5	24.0	47.5
(b) Loose	99.5	86.3	86.2	53.6	76.5

^a^ Two faulty measurements were excluded from the calculations (*n* = 181). ^b^ Matching was based on the minimal *ΔE_00_* to the *labeled* RAL+ colors. ^c^ Matching accuracy when 19 missing RAL+ *assessed* colors were dismissed.

**Table 3:** Color differences (*ΔE_00_* median and, below in the squared brackets, minimum, 25th, 75th percentile, maximum) for (a) *measured–labeled*, (b) *measured–assessed*, and (c) *normalize–labeled* RAL+ colors (*labeled*: RAL+ color label, *assessed*: evaluated by the device manufacturers, *measured*: estimated by the spectrometers, *normalized*: mapped to the *labeled* RAL+ colors).

Comparison	Nix Spectro 2	Spectro 1 Pro	ColorReader	Pico	Smartphone
(a) *Measured* vs. labeled *	1.87 [0.38 1.32 2.42 4.34]	1.79 [0.42 1.04 3.59 9.41]	1.27 [0.33 0.84 1.83 7.19]	3.95 [1.23 2.64 5.88 12.84]	/ ^b^
(b) *Measured* vs. *assessed*	0.52 [0.16 0.43 0.66 1.30]	1.07 [0.21 0.74 1.53 3.63]	1.39 [0.01 0.98 1.83 4.31]	/ ^a^	/ ^b^
(c) *Normalized* vs. *labeled*	1.01 [0.19 0.73 1.43 3.83]	1.19 [0.14 0.86 1.60 7.14]	1.11 [0.20 0.77 1.61 6.33]	1.88 [0.26 1.23 2.85 7.37]	1.84 [0.20 1.21 3.21 8.51]

* The difference between *measured* and *labeled* colors is a limited measure for colorimetric accuracy (see Section 2.4), ^a^ Pico does not provide *assessed* RAL+ color values. ^b^ Not applicable.

**Table 4:** Overall colorimetric performances (matching and evaluating RAL+ colors in the CIELAB color space) and listed prices of four spectrophotometers and a smartphone used in this study.

Device	RAL+ Color Matching	*ΔE_00_* ^a^	Price
(1) Nix Spectro 2	almost perfect (~99%)	0.52–1.05	~USD 1200
(2) Spectro 1 Pro, ColorReader	very good (~85%)	1.07–1.39	USD 130–300
(3) Pico, smartphone Asus 8	good (~54–76%)	~1.85	USD 120–400 ^b^

^a^ Estimated from matched, *assessed*, and *normalized* RAL+ colors (Section 3.1 and Section 3.2). ^b^ USD 400 is the price of dermatoscope DL1, needed for colorimetric calibration of a smartphone.

## Data Availability

The raw data supporting the conclusions of this article will be made available by the authors on request.
